# Osteoporosis Complicating Renal Tubular Acidosis in Association With Sjogren's Syndrome

**DOI:** 10.7759/cureus.18373

**Published:** 2021-09-29

**Authors:** Saira Furqan, Sabiha Banu, Nanik Ram

**Affiliations:** 1 Endocrinology, Aga Khan University Hospital, Karachi, PAK

**Keywords:** osteoporosis, primary hypothyroidism, fracture, hypokalemic periodic paralysis, metabolic acidosis

## Abstract

Chronic metabolic acidosis increases alkali mobilization from the bone and promotes the development of osteoporosis. We report the case of a 35-year-old Caucasian female who presented for surgical fixation of a left femoral fracture sustained six months previously from a ground level fall with known primary hypothyroidism (for 12 years, on thyroxine replacement) with history of hypokalemia for the last 13 years (on regular oral potassium supplements). There was no history of fracture in past. There was no history of renal stones. There was positive history of hypokalemic periodic paralysis twice in past (12 and 13 years back). There was no history of recurrent oral or ocular ulcers. On examination there was no uveitis, oral ulcers, lacrimal or parotid gland enlargement. Upon workup the patient was diagnosed with left-sided femur fracture (neck) and was admitted for surgical management. She underwent left dynamic hip screw fixation under general anesthesia which she tolerated well. Upon further workup she had normal anion gap with hyperchloremic metabolic acidosis, low vitamin D level and dual-energy x-ray absorptiometry (DEXA) scan revealed osteoporosis at femur and hip joint. Vitamin D was replaced, she was started on Ibandronate and calcium supplements. Her further workup revealed positive anti-SSA. Our final clinical diagnosis in this case is possible or incomplete Sjogren's syndrome causing type-1 renal tu­bular acidosis (RTA; distal RTA) with osteoporosis.

## Introduction

Sjogren's syndrome (SS) is a chronic disorder of the immune system involving exocrine glands. It causes lymphocytic infiltrates in the salivary glands [1]. Most people are older than 40 years of age at the time of diagnosis. Xerostomia and xerophthalmia are the predominant clinical features in adults. There are two main types of extraglandular involvement in Sjogren's syndrome: extraepithelial extraglandular involvement and periepithelial infiltrative involvement. Extraepithelial extraglandular involvement in Sjögren's syndrome is linked to hypergammaglobulinemia, B-cell hyperreactivity and immune complex formation and includes peripheral neuropathy, palpable purpura and glomerulonephritis. Periepithelial infiltrative processes usually follow a benign course and include hepatic involvement, interstitial nephritis, and bronchiolitis. The most prevalent renal involvement in Sjogren's syndrome is tubulointerstitial nephritis, leading to renal tu­bular acidosis (RTA) in 20% of patients [2]. There are three types of renal tubular acidosis: type-1 RTA, type-2 RTA and type-4 RTA. 

Type-1 distal RTA (RTA type-1) is marked by non-anion gap hyperchloremic metabolic acidosis and hypokalemia. Type-2 proximal RTA (RTA type-2) may occur due to gene­ralized dysfunction of the proximal tubules and is associated with increased urinary excre­tion of phosphate, amino acids, glucose, uric acid and proteins [3]. Type-4 renal tubular acidosis (RTA) is characterized by metabolic acidosis with persistent hyperkalemia that occurs usually in patients suffering from mild to moderate chronic glomerular insufficiency. Osteoporosis is rarely encountered in clinical practice as the first manifestation of renal tubule dis­order secondary to Sjogren’s syndrome [4].

## Case presentation

A 35-year-old Caucasian female patient who presented for surgical fixation of left femur fracture sustained six months previously from a ground level fall with known primary hypothyroidism (for 12 years, on thyroxine replacement) with history of hypokalemia for last 13 years (on regular oral potassium supplements). Upon workup the patient was diagnosed with left-sided neck of femur fracture and was admitted in hospital for surgical management. There was no history of fracture in past. Her menstrual cycle was regular. There was no history of renal stones. There was positive history of hypokalemic periodic paralysis twice in past (12 and 13 years back). Her family history was unremarkable. There was no history of recurrent oral or ocular ulcers. On examination there was no uveitis, oral ulcers, lacrimal or parotid gland enlargement. Her workup was done which is shown in Table 1 below. It showed hyperchloremic non anion gap metabolic acidosis and hypokalemia. DEXA scan revealed osteoporosis at right femoral neck (both T and Z scores were <-2.5) and at right hip (both T and Z scores were <-2.5).

**Table 1:** Investigations WBC, White blood cell. Urine DR, Urine detailed report. TSH, Thyroid stimulating hormone. ESR, Erythrocyte sedimentation rate. PTH, Parathyroid hormone. ANA, Antinuclear antigen. DEXA, Dual energy x-ray absorptiometry.

Name of investigation	Patient’s results	Normal ranges
Hemoglobin	11.4	11.1-14.5 gm/dl
WBC	8.4	4.0-10.0*10E9/L
Platelets	513	150-400*10E9/L
Sodium	139	136-145 mmol/l
Potassium	3.1	3.2-5.1 mmol/l
Chloride	115	98-107 mmol/l
Bicarbonate	13.6	20-31 mmol/l
Urine DR	PH : 7.0 , specific gravity: 1.010 Protein: 1+ , Glucose : - ve	
TSH	9.97	0.4-4 u IU/ml
Creatinine	0.91	
ESR	75	0-20 mm/1^st^ hr
Vitamin D level	18.8	>30 ng/ml
PTH	54.7	16-87 pg/ml
Calcium	8.4	8.6-10.2 mg/dl
Phosphorus	1.9	2.5-4.5mg/dl
Albumin	4.3	3.5-5.2 g/dl
ANA	Negative	
DEXA scan	Right hip T score : -3.2 Z score : -3.0 Right femoral neck T score : -3.6 Z score : -3.3 Spine (L1-L4 ) T score : 0.1 Right Distal forearm T score : 0.1	

She underwent left-sided dynamic hip screw fixation under general anesthesia which she tolerated well. On the fourth postoperative day she was allowed full weight bearing with walker. After surgery she was started on oral Ibandronate 150 mg once monthly (for osteoporosis) and tablet calcium carbonate (600 mg elemental calcium) twice daily and vitamin D 2000 IU twice daily. Due to low vitamin D levels she was given one intramuscular injection of vitamin D (200,000 IU). Oral potassium supplement three tablets thrice daily were continued. Potassium citrate was added in her regimen to correct renal tubular acidosis. Thyroxine 100 mcg was continued five days a week and 150 mcg was advised two days a week (the dose was adjusted according to recent thyroid stimulating hormone (TSH) value of 9.97 which is suboptimal). So the possible clinical diagnosis in this case is renal tubular acidosis with osteoporosis and hypokalemia. Nephrology recommended anti-SSA and anti-SSB despite negative antinuclear antibody (ANA) screen as these may be positive in RTA. In this patient anti-SSA came positive while anti-SSB came negative. During follow up her potassium level got normalized to 4.6, bicarbonate improved to 20.2 and TSH normalized to 1.45. We will get her repeat DEXA scan next month (to see any improvement in her bone mineral density after correction of vitamin D deficiency). Our clinical diagnosis in this patient is possible or incomplete Sjogren's syndrome (as she lacks few clinical features on examination) causing type-1 RTA (distal RTA) with osteoporosis.

## Discussion

Overt or latent RTA caused secondary to autoimmune tubulointerstitial nephropathy is frequently encountered extra-glandular manifestation of Sjogren's syndrome and is observed in 33% of cases [5]. The underlying defect is deficient H+-ATPase pump function [6]. There is one case report published in Saudi Journal of Kidney Diseases and Transplantation in 2007 with the title *Osteomalacia complicating renal tubular acidosis in association with Sjogren's syndrome* [7].

The peculiar feature of RTA is the presence of normal anion gap with hyperchloremic metabolic acidosis. The serum potassium may be low, high or normal depending on the type of renal tubular acidosis. Sjogren's syndrome is most frequently encountered with type-2 RTA. Type-1 RTA is also called "distal RTA" as presence of a huge pH gradient between blood and urine is the function of distal nephron. Our patient had a normal anion gap, hypokalemia, hyperchloremic acidosis and alkaline urine in addition to sys­temic acidosis confirming the diagnosis of type-1 RTA. Proximal RTA (type-2 RTA) is due to inability of reabsorption of bicarbonate by the proximal tubules. It may occur secondary to generalized dysfunction of the proximal tu­bules and is associated with increased excretion of amino acids, proteins, glucose, uric acid and phos­phate in urine, as shown in Table 2 [3,8]. The proximal tubule dysfunction most often occurs in patients with light chain nephropathy, multiple mye­loma, Fanconi syn­drome or drug exposure. Table 2 highlights the important differences in different types of renal tubular acidosis which helps in deciding which type of RTA the patient has.

**Table 2:** Distinguishing features of different types of RTA HCO3, Bicarbonate.

	RTA type 1	RTA type 2	RTA type 4
Primary defect	Impaired ability to excrete H+ in distal tubule	Impaired HCO3 reabsorption in proximal tubule	Decreased secretion of aldosterone or decreased defect
Minimum Urine PH	PH > 5.5	PH < 5.5	PH < 5.5
Stones	Yes	No	No
Hyperchloremic acidosis	Yes	Yes	Yes
Serum Potassium	Low-normal	Low- normal	High
Plasma Bicarbonate	<10 meq/L	12-20 meq/L	>17 meq/L

Osteomalacia caused by RTA is rarely seen as the presenting feature of Sjogren's syndrome. It is far more common with proximal than with distal RTA [4]. In proximal RTA, renal phosphate loss is the main contributing factor to osteomalacia, while in distal RTA it is secondary to hypophosphatemia and acidosis and in addition associated vitamin D deficiency may be another contributing factor.

## Conclusions

Renal tubular acidosis has been identified to lead to osteomalacia in adults. Bone involvement is more frequent in proximal RTA (type 2) but distal RTA (type 1) can also cause osteomalacia and osteoporosis due to loss of calcium salts from bone and hypophosphatemia. Our case is rare, unique and interesting because in spite of the rarity of osteoporosis revealing Sjogren’s syndrome, this compli­cation should be taken into consideration by physicians in the diagnosis of Sjogren’s syndrome with renal tubular acidosis. Latent renal tubular disease is quite prevalent in Sjogren’s syndrome, but is infrequently complicated by osteoporosis. Pri­mary Sjogren’s syndrome could be a differential diagnosis in patients with muscular weakness, mild hypokalemia and osteoporosis.
